# Supplementary material for: Pentoxifylline in the Treatment of Cutaneous Leishmaniasis: A Randomized Clinical Trial in Colombia
Source: Pathogens. 2022 Mar 21;11(3):378. doi: 10.3390/pathogens11030378 (PMC8949591; doi:10.3390/pathogens11030378)
Supplement: Supplementary file 1 [file pathogens-11-00378-s001.zip › pathogens-1617324-supplementary.pdf]

Supplementary material:

Table S1. Therapeutic Response by Time of Follow-up.

		PTX + MA (n=34) n (%)	Placebo + MA (n=36) n (%)	p^*
**End of treatment (Day 21)**	Apparent cure	0 (0.00)	1 (2.78)	0.480
	Improvement	33 (97.06)	31 (86.11)	
	Therapeutic failure in Visit 2	0 (0.00)	1 (2.78)	
	Did not attend the visit	1 (2.94)	3 (8.33)	
**Week 5 (Day 35)**	Apparent cure	1 (2.94)	6 (16.67)	0.124
	Improvement	30 (88.24)	23 (63.89)	
	Without changes	1 (2.94)	2 (5.56)	
	Therapeutic failure in another visit	0 (0.00)	1 (2.78)	
	Did not attend the visit	2 (5.88)	4 (11.11)	
**Week 7 (Day 49)**	Apparent cure	7 (20.59)	14 (38.89)	0.074
	Improvement	17 (50.00)	18 (50.00)	
	Without changes	6 (17.65)	2 (5.56)	
	Therapeutic failure in visit 4	0 (0.00)	1 (2.78)	
	Therapeutic failure at another visit	0 (0.00)	1 (2.78)	
	Did not attend the visit	2 (5.88)	0 (0.00)	
	Lost to follow up in Visit 4	2 (5.88)	0 (0.00)	
**Week 13 (Day 90)**	Apparent cure	18 (52.94)	20 (55.56)	0.580
	Improvement	6 (17.65)	5 (13.89)	
	Without changes	0 (0.00)	1 (2.78)	
	Therapeutic failure in Visit 5	7 (20.59)	5 (13.89)	
	Therapeutic failure in another visit	0 (0.00)	2 (5.56)	
	Did not attend the visit	1 (2.94)	2 (5.56)	
	Lost to follow up in Visit 5	0 (0.00)	1 (2.78)	
	Lost to follow up in another visit	2 (5.88)	0 (0.00)	
**Week 26 (Day 180)**	Definitive cure	22(64.71)	27 (75.00)	0.690
	Therapeutic failure in visit 6	2 (5.88)	1 (2.78)	
	Therapeutic failure in another visit	7 (20.59)	7 (19.44)	
	Lost to follow up in Visit 6	1 (2.94)	0 (0.00)	
	Lost to follow up in another visit	2 (5.88)	1 (2.78)	

* Fisher´s exact test

Table S2. List of 84 Genes Included in the Expression Analysis.

Symbol	Description
bcl6	B-cell CLL/lymphoma 6
c3	Complement component 3
c3ar1	Complement component 3a receptor 1
*ccl11**	Chemokine (C-C motif) ligand 11
*ccl13**	Chemokine (C-C motif) ligand 13
*ccl16**	Chemokine (C-C motif) ligand 16
*ccl17**	Chemokine (C-C motif) ligand 17
*ccl19**	Chemokine (C-C motif) ligand 19
ccl2	Chemokine (C-C motif) ligand 2
*ccl21**	Chemokine (C-C motif) ligand 21
ccl22	Chemokine (C-C motif) ligand 22
*ccl23**	Chemokine (C-C motif) ligand 23
ccl24	Chemokine (C-C motif) ligand 24
ccl3	Chemokine (C-C motif) ligand 3
ccl4	Chemokine (C-C motif) ligand 4
ccl5	Chemokine (C-C motif) ligand 5
ccl7	Chemokine (C-C motif) ligand 7
*ccl8**	Chemokine (C-C motif) ligand 8
ccr1	Chemokine (C-C motif) receptor 1
ccr2	Chemokine (C-C motif) receptor 2
*ccr3**	Chemokine (C-C motif) receptor 3
ccr4	Chemokine (C-C motif) receptor 4
ccr7	Chemokine (C-C motif) receptor 7
cd14	CD14 molecule
cd40	CD40 molecule, TNF receptor superfamily member 5
cd40lg	CD40 ligand
cebpb	CCAAT/enhancer binding protein (C/EBP), beta
*crp**	C-reactive protein, pentraxin-related
csf1	Colony stimulating factor 1 (macrophage)
cxcl1	Chemokine (C-X-C motif) ligand 1 (melanoma growth stimulating activity, alpha)
cxcl10	Chemokine (C-X-C motif) ligand 10
cxcl2	Chemokine (C-X-C motif) ligand 2
cxcl3	Chemokine (C-X-C motif) ligand 3
cxcl5	Chemokine (C-X-C motif) ligand 5
*cxcl6**	Chemokine (C-X-C motif) ligand 6 (granulocyte chemotactic protein 2)
cxcl9	Chemokine (C-X-C motif) ligand 9
*cxcr1**	Chemokine (C-X-C motif) receptor 1
cxcr2	Chemokine (C-X-C motif) receptor 2
cxcr4	Chemokine (C-X-C motif) receptor 4
faslg	Fas ligand (TNF superfamily, member 6)
fos	FBJ murine osteosarcoma viral oncogene homolog
ifng	Interferon, gamma
il10	Interleukin 10
il10rb	Interleukin 10 receptor, beta
il15	Interleukin 15
*il17a**	Interleukin 17A
*il18**	Interleukin 18 (interferon-gamma-inducing factor)
il1a	Interleukin 1, alpha
il1b	Interleukin 1, beta
il1r1	Interleukin 1 receptor, type I
il1rap	Interleukin 1 receptor accessory protein

il1rn	Interleukin 1 receptor antagonist
*il22**	Interleukin 22
il23a	Interleukin 23, alpha subunit p19
*il23r**	Interleukin 23 receptor
il5	Interleukin 5 (colony-stimulating factor, eosinophil)
il6	Interleukin 6 (interferon, beta 2)
il6r	Interleukin 6 receptor
il8	Interleukin 8
*il9**	Interleukin 9
itgb2	Integrin, beta 2 (complement component 3 receptor 3 and 4 subunit)
*kng1**	Kininogen 1
lta	Lymphotoxin alpha (TNF superfamily, member 1)
ltb	Lymphotoxin beta (TNF superfamily, member 3)
ly96	Lymphocyte antigen 96
myd88	Myeloid differentiation primary response gene (88)
nfkb1	Nuclear factor of kappa light polypeptide gene enhancer in B-cells 1
*nos2**	Nitric oxide synthase 2, inducible
nr3c1	Nuclear receptor subfamily 3, group C, member 1 (glucocorticoid receptor)
ptgs2	Prostaglandin-endoperoxide synthase 2 (prostaglandin G/H synthase and cyclooxygenase)
ripk2	Receptor-interacting serine-threonine kinase 2
*sele**	Selectin E
tirap	Toll-interleukin 1 receptor (TIR) domain containing adaptor protein
tlr1	Toll-like receptor 1
tlr2	Toll-like receptor 2
tlr3	Toll-like receptor 3
tlr4	Toll-like receptor 4
tlr5	Toll-like receptor 5
tlr6	Toll-like receptor 6
tlr7	Toll-like receptor 7
*tlr9**	Toll-like receptor 9
tnf	Tumor necrosis factor
*tnfsf14**	Tumor necrosis factor (ligand) superfamily, member 14
tollip	Toll interacting protein

* Not detected

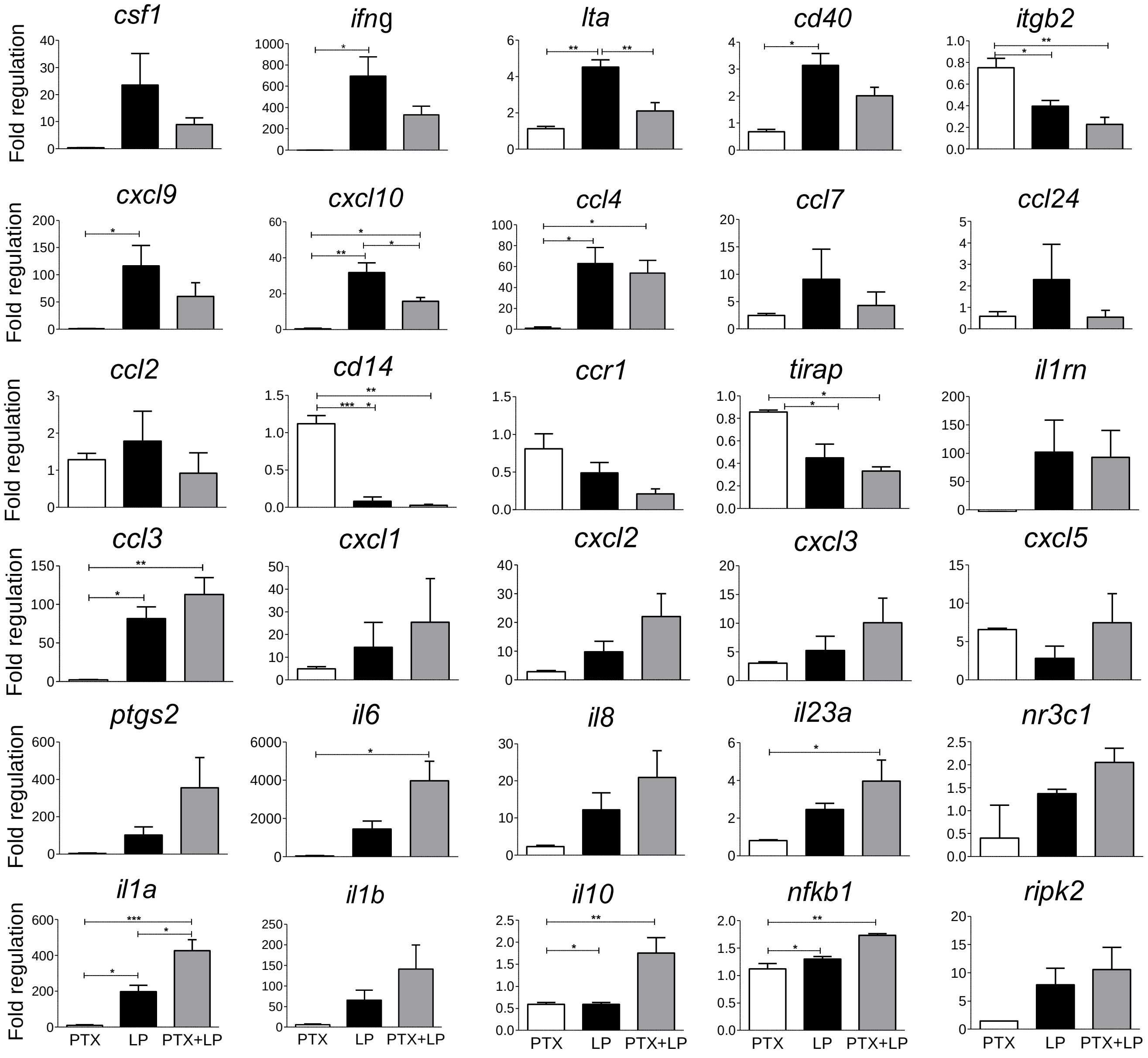

Figure S1. Modulation of gene expression by pentoxifylline during *ex vivo* infection with *L. (V.) panamensis*. PBMCs obtained before treatment from patients with active CL (n = 3) were exposed to 200 µM of PTX, *L. (V.) panamensis* (LP), or Lp+200 µM of PTX (PTX+LP). The genes presented correspond to those that were significantly modulated or showed a notable but not statistically significant modulation. Data are presented as the mean ± SEM of fold regulation compared with PBMCs without *ex vivo* treatment. One-way ANOVA test was used to establish statistical differences among groups and Tukey's multiple comparison test was performed on subsets of data. *p < 0.05, **p < 0.01.

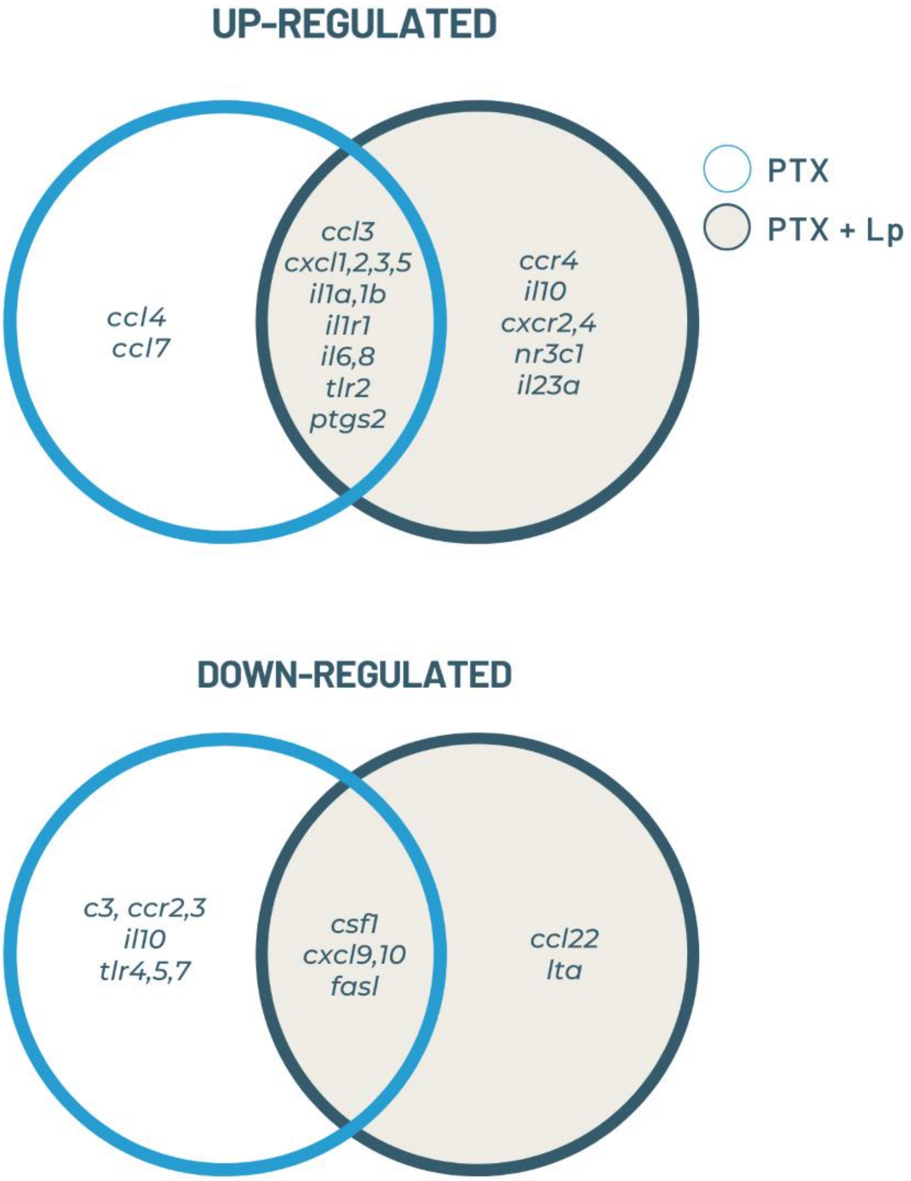

Figure S2. Schematic representation of immune mediators modulated by PTX in both uninfected or infected PBMCs obtained before treatment from patients with CL. Genes upregulated (A) or downregulated (B) compared with PBMCs not exposed to PTX.
